# Validation of the orthopedic frailty score for measuring frailty in hip fracture patients: a cohort study based on the United States National inpatient sample

**DOI:** 10.1007/s00068-023-02308-7

**Published:** 2023-06-22

**Authors:** Maximilian Peter Forssten, Yang Cao, Ahmad Mohammad Ismail, Ioannis Ioannidis, Lakshika Tennakoon, David A. Spain, Shahin Mohseni

**Affiliations:** 1 Department of Orthopedic Surgery, Orebro University Hospital, 701 85 Orebro, Sweden; 2 School of Medical Sciences, Orebro University, 702 81 Orebro, Sweden; 3 Clinical Epidemiology and Biostatistics, School of Medical Sciences, Faculty of Medicine and Health, Orebro University, 701 82 Orebro, Sweden; 4 Department of Surgery, Section of Trauma and Acute Care Surgery, Stanford University School of Medicine, Stanford, CA USA; 5 Division of Trauma, Critical Care and Acute Care Surgery, Department of Surgery, Sheik Shakhbout Medical City – Mayo Clinic, Abu Dhabi, United Arab Emirates

**Keywords:** Hip fracture, Orthopedic frailty score, Mortality, Complication, Risk stratification

## Abstract

**Background:**

The Orthopedic Frailty Score (OFS) has been proposed as a tool for measuring frailty in order to predict short-term postoperative mortality in hip fracture patients. This study aims to validate the OFS using a large national patient register to determine its relationship with adverse outcomes as well as length of stay and cost of hospital stay.

**Methods:**

All adult patients (18 years or older) registered in the 2019 National Inpatient Sample Database who underwent emergency hip fracture surgery following a traumatic fall were eligible for inclusion. The association between the OFS and mortality, complications, and failure-to-rescue (FTR) was determined using Poisson regression models adjusted for potential confounders. The relationship between the OFS and length of stay and cost of hospital stay was instead determined using a quantile regression model.

**Results:**

An estimated 227,850 cases met the study inclusion criteria. There was a stepwise increase in the rate of complications, mortality, and FTR for each additional point on the OFS. After adjusting for potential confounding, OFS 4 was associated with an almost ten-fold increase in the risk of in-hospital mortality [adjusted IRR (95% CI): 10.6 (4.02–27.7), p < 0.001], a 38% increased risk of complications [adjusted IRR (95% CI): 1.38 (1.03–1.85), p = 0.032], and an almost 11-fold increase in the risk of FTR [adjusted IRR (95% CI): 11.6 (4.36–30.9), p < 0.001], compared to OFS 0. Patients with OFS 4 also required a day and a half additional care [change in median length of stay (95% CI): 1.52 (0.97–2.08), p < 0.001] as well as cost approximately $5,200 more to manage [change in median cost of stay (95% CI): 5166 (1921–8411), p = 0.002], compared to those with OFS 0.

**Conclusion:**

Patients with an elevated OFS display a substantially increased risk of mortality, complications, and failure-to-rescue as well as a prolonged and more costly hospital stay.

## Introduction

The population of hip fracture patients presents a major challenge for healthcare systems worldwide due to their high degree of frailty and significant comorbidity burden [1–3]. With up to 50% of hip fracture patients being classified as frail [4, 5], frailty is likely a leading cause of the high postoperative mortality rates observed in hip fracture patients [6, 7]. Approximately 2.7 million people suffered a hip fracture during 2010, comprising around 20% of all osteoporotic fractures among individuals 50 years or older [8–10]. This number is projected to grow as the global population ages and life expectancy increases, putting further strain on hospital and public resources [11–15].

The estimation of postoperative mortality risk can be a critical aspect of patient care and is important for tailoring preoperative and postoperative care, surgical management, resource allocation, and facilitating discussions between patients, their families, and healthcare providers regarding treatment plans and expectations [16]. The Orthopedic Frailty Score (OFS) has been proposed as potential tool for filling this role. Developed with the intention of predicting short-term mortality in hip fracture patients, the OFS makes use of five easily obtainable variables from the patient’s history or records to estimate the patient’s degree of frailty [17]. However, previous investigations have chiefly focused on its relationship with 30-day and 90-day postoperative mortality in Swedish datasets [5, 17, 18]. The aim of this study is therefore to validate the OFS using a large national American patient register in order to determine the relationship between the OFS and in-hospital mortality, complications, failure-to-rescue, as well as length of stay and cost of hospital stay.

## Methods

The study adhered to the principles of the Declaration of Helsinki as well as the STROBE guidelines [19], and received ethical approval from the Swedish Ethical Review Authority (ref: 2022–03107-02). The data for the current investigation was obtained from the 2019 United States National Inpatient Sample (NIS). The NIS is maintained by the Agency for Healthcare Research and Quality and is a part of the Healthcare Cost and Utilization Project (HCUP), which is the largest all-payer inpatient database in the United States, sampling 20% of all hospitalizations in the country. The study employed validated sampling algorithms with discharge and survey weights to provide accurate national estimates for 97% of all inpatient hospitalizations in the United States [20]. In accordance with prior studies, using International Classification of Diseases 10th Revision (ICD-10) codes, all adult patients (18 years or older) registered in the NIS who underwent emergency hip fracture surgery subsequent to a traumatic fall using internal fixation (open reduction internal fixation or intramedullary nailing) or arthroplasty (total hip arthroplasty or hemiarthroplasty) were included [21]. Patients with head, vascular, or truncal injuries were excluded in order to reduce the heterogeneity in the dataset [21]. Patients were also excluded if they were missing variables needed to calculate the OFS. Data retrieved included patient demographics, such as age, sex, race/ethnicity, socioeconomic status, comorbidities, clinical characteristics of the injury and management strategy, as well as outcomes including in-hospital mortality, complications [22], and failure-to-rescue (FTR). The Elixhauser Comorbidity Index was calculated using 38 different pre-existing conditions listed on hospital administrative data using the Elixhauser Comorbidity Software Refined for ICD-10-CM developed as part of the HCUP [23, 24].

### Calculating the orthopedic frailty score

The OFS was specifically developed for the purpose of evaluating frailty and predicting short-term mortality, up to 90-days postoperatively, among patients who have suffered a hip fracture. It is determined by assessing the presence of five dichotomous variables: congestive heart failure, a history of malignancy (excluding non-invasive skin cancer), institutionalization, dependence on assistance for activities of daily living (non-independent functional status), and an age of 85 years or older. Each variable is assigned one point, resulting in a maximum possible score of 5 [17]. Those with OFS 0 are considered non-frail, those with OFS 1 as pre-frail, and those with OFS ≥ 2 are classified as frail [5, 17]. Frail patients have previously been found to suffer from a 4 times as high risk of 30-day and 90-day postoperative mortality as non-frail hip fracture patients [5].

### Statistical analysis

As in previous investigations into the role of risk stratification tools in hip fracture patients, the current study divided patients into five groups based on their OFS: OFS 0, 1, 2, 3, and 4. No patient had an OFS of 5 in the current dataset. A comparison of patient demographics, clinical characteristics, and outcomes was conducted between the groups under examination. The data pertaining to categorical variables were presented in terms of counts and percentages, while continuous variables were reported as the median and interquartile range. To assess the statistical significance of any differences between categorical variables, the Chi-squared test was employed. For continuous variables the Kruskal–Wallis test was employed instead. The primary outcome of interest was in-hospital mortality, with secondary outcomes of interest consisting of complications (any cardiac complication, heart failure, cardiac arrest, cardiac tamponade, ventricular tachycardia, ventricular fibrillation, stroke, acute kidney injury, delirium, pulmonary embolism, deep vein thrombosis, embolism due to orthopedic device, sepsis, urinary tract infection, pneumonia, wound infection, implant infection, decubitus ulcer), FTR (defined as in-hospital mortality subsequent to a complication), length of stay (in days), and total cost of hospital stay (in United States dollars).

Poisson regression models were employed to determine the association between the OFS and in-hospital mortality, complications as well as FTR. To minimize the bias due to confounding, all analyses were adjusted for potential confounders, including age, sex, race/ethnicity, income quartile, comorbidities (ischemic heart disease, cerebrovascular disease, peripheral vascular disease, uncomplicated diabetes mellitus, diabetes mellitus with chronic complications, end stage renal disease, dementia, connective tissue disease, hypothyroidism, liver disease, coagulopathy, obesity, fluid or electrolyte disorders, chronic obstructive pulmonary disease, alcohol abuse, drug abuse, psychoses, depression, osteoporosis, osteoporosis with fracture) smoking status, type of fracture, and type of surgery. Due to the generally short length of stay, no correction was required for time dependence in the Poisson regression analysis. The associations are reported as incidence rate ratios (IRRs) with 95% confidence intervals (CIs) calculated using robust standard errors [25].

Quantile regression models were instead used to examine the effects of the OFS on the length of stay as well as total cost of hospital stay. The same covariates were included in this analysis as in the aforementioned Poisson regression models, and the outcomes were presented as the change in the median length of stay or total cost, accompanied by the corresponding 95% CIs.

A two-sided p-value less than 0.05 was considered statistically significant in all analyses. As 4% of cases (N = 9,100) were missing data, multiple imputation by chained equations was employed [26]. All analyses were performed in the software R 4.0.5 using packages *tidyverse*, *haven*, *mice*, *survey*, and *quantreg* [27].

## Results

Of the 253,135 surgically managed hip fracture cases registered in the NIS, 24,415 were excluded due the presence of a head, vascular, or truncal injury, and 870 were excluded due to a missing OFS, resulting in an estimated 227,850 cases being included for further analysis. As shown in Table 1, as the OFS increased, the patients’ median age tended to increase (OFS 0 vs. OFS 4: 74 years vs 90 years, p < 0.001). The majority of all patients were white, while those with a higher OFS tended to be more affluent (OFS 0 vs. OFS 4, 76th to 100th income percentile: 20.3% vs 32.0%, p < 0.001) and suffer from a higher comorbidity burden according to their Elixhauser Comorbidity Index (OFS 0 vs. OFS 4: 2 vs 4, p < 0.001). Most patients suffered a cervical or pertrochanteric hip fracture and internal fixation was the most common management strategy (Table 1). The majority of comorbidities increased in prevalence along with the OFS except for diabetes mellitus, liver disease, obesity, substance abuse, psychiatric diagnoses, chronic obstructive pulmonary disease, previous fracture due to osteoporosis, fluid or electrolyte disorders, and connective tissue disease (Table 2).

**Table 1:** Demographics of hip fracture patients

	OFS 0 (N = 109,410)	OFS 1 (N = 87,115)	OFS 2 (N = 27,755)	OFS 3 (N = 3,320)	OFS 4 (N = 250)	P-value
Age, median [IQR]	74 [67.0–80.0]	87 [80.0–90.0]	89 [86.0–90.0]	90 [87.0–90.0]	90 [87.0–90.0]	< 0.001
Sex, n (%)						< 0.001
Male	35,370 (32.3)	26,065 (29.9)	8,505 (30.6)	1,050 (31.6)	85 (34.0)	
Female	74,040 (67.7)	61,050 (70.1)	19,250 (69.4)	2,270 (68.4)	165 (66.0)	
Race/Ethnicity, n (%)						< 0.001
White	91,750 (83.9)	73,440 (84.3)	23,995 (86.5)	2,900 (87.3)	215 (86.0)	
Black	4,590 (4.2)	3,460 (4.0)	1,045 (3.8)	125 (3.8)	10 (4.0)	
Hispanic	6,115 (5.6)	4,795 (5.5)	1,195 (4.3)	125 (3.8)	5 (2.0)	
Asian or Pacific Islander	1,860 (1.7)	1,605 (1.8)	440 (1.6)	30 (0.9)	15 (6.0)	
American Indian	560 (0.5)	325 (0.4)	80 (0.3)	5 (0.2)	5 (2.0)	
Other	2,165 (2.0)	1,680 (1.9)	500 (1.8)	60 (1.8)	0 (0.0)	
Missing	2,370 (2.2)	1,810 (2.1)	500 (1.8)	75 (2.3)	0 (0.0)	
Income quartile, n (%)						< 0.001
0–25th percentile	29,635 (27.1)	22,240 (25.5)	6,260 (22.6)	685 (20.6)	25 (10.0)	
26th to 50th percentile	28,400 (26.0)	22,875 (26.3)	6,530 (23.5)	765 (23.0)	65 (26.0)	
51st to 75th percentile	27,270 (24.9)	21,505 (24.7)	7,345 (26.5)	980 (29.5)	75 (30.0)	
76th to 100th percentile	22,240 (20.3)	19,335 (22.2)	7,305 (26.3)	875 (26.4)	80 (32.0)	
Missing	1,865 (1.7)	1,160 (1.3)	315 (1.1)	15 (0.5)	5 (2.0)	
Elixhauser Comorbidity Index, median [IQR]	2 [1.0–3.0]	3 [2.0–4.0]	3 [2.0–4.0]	4 [3.0–5.0]	4 [2.5–4.5]	< 0.001
Type of fracture, n (%)						< 0.001
Cervical	54,415 (49.7)	39,480 (45.3)	12,215 (44.0)	1,425 (42.9)	130 (52.0)	
Basicervical	1,865 (1.7)	1,505 (1.7)	370 (1.3)	40 (1.2)	0 (0.0)	
Pertrochanteric	48,115 (44.0)	42,595 (48.9)	14,105 (50.8)	1,720 (51.8)	110 (44.0)	
Subtrochanteric	4,970 (4.5)	3,485 (4.0)	1,060 (3.8)	130 (3.9)	10 (4.0)	
Missing	45 (0.0)	50 (0.1)	5 (0.0)	5 (0.2)	0 (0.0)	
Type of surgery, n (%)						0.096
Internal fixation	68,920 (63.0)	55,270 (63.4)	17,935 (64.6)	2,195 (66.1)	145 (58.0)	
Arthroplasty	40,490 (37.0)	31,845 (36.6)	9,820 (35.4)	1,125 (33.9)	105 (42.0)	

*OFS, Orthopedic Frailty Score*

**Table 2:** Comorbidities in hip fracture patients

	OFS 0 (N = 109,410)	OFS 1 (N = 87,115)	OFS 2 (N = 27,755)	OFS 3 (N = 3,320)	OFS 4 (N = 250)	P-value
Ischemic heart disease, n (%)	19,425 (17.8)	25,350 (29.1)	11,090 (40.0)	1,405 (42.3)	95 (38.0)	< 0.001
Congestive heart failure, n (%)	0 (0.0)	15,930 (18.3)	17,420 (62.8)	2,715 (81.8)	240 (96.0)	< 0.001
Cerebrovascular disease, n (%)	6,360 (5.8)	5,690 (6.5)	1,965 (7.1)	250 (7.5)	15 (6.0)	0.001
Peripheral vascular disease, n (%)	4,715 (4.3)	4,225 (4.8)	1,500 (5.4)	160 (4.8)	10 (4.0)	0.006
Uncomplicated diabetes mellitus, n (%)	11,270 (10.3)	7,070 (8.1)	1,735 (6.3)	245 (7.4)	0 (0.0)	< 0.001
Diabetes mellitus with chronic complications, n (%)	13,410 (12.3)	9,965 (11.4)	3,470 (12.5)	405 (12.2)	25 (10.0)	0.089
End stage renal disease, n (%)	11,490 (10.5)	16,160 (18.6)	6,850 (24.7)	845 (25.5)	45 (18.0)	< 0.001
Liver disease, n (%)	3,155 (2.9)	1,130 (1.3)	180 (0.6)	40 (1.2)	0 (0.0)	< 0.001
Coagulopathy, n (%)	5,525 (5.0)	5,115 (5.9)	1,555 (5.6)	185 (5.6)	15 (6.0)	0.011
Obese, n (%)	6,650 (6.1)	2,625 (3.0)	725 (2.6)	65 (2.0)	10 (4.0)	< 0.001
Alcohol abuse, n (%)	6,320 (5.8)	1,190 (1.4)	120 (0.4)	20 (0.6)	0 (0.0)	< 0.001
Drug abuse, n (%)	2,250 (2.1)	630 (0.7)	125 (0.5)	10 (0.3)	0 (0.0)	< 0.001
Psychosis, n (%)	3,765 (3.4)	1,545 (1.8)	405 (1.5)	85 (2.6)	5 (2.0)	< 0.001
Depression, n (%)	14,125 (12.9)	8,515 (9.8)	2,385 (8.6)	255 (7.7)	15 (6.0)	< 0.001
Current smoker, n (%)	925 (0.8)	420 (0.5)	95 (0.3)	15 (0.5)	5 (2.0)	< 0.001
Chronic obstructive pulmonary disease, n (%)	2,790 (2.6)	2,025 (2.3)	640 (2.3)	70 (2.1)	10 (4.0)	0.506
Dementia, n (%)	17,085 (15.6)	27,970 (32.1)	11,310 (40.7)	1,675 (50.5)	140 (56.0)	< 0.001
Non-independent functional status, n (%)	0 (0.0)	4,690 (5.4)	4,515 (16.3)	1,405 (42.3)	185 (74.0)	< 0.001
Institutionalized, n (%)	0 (0.0)	4,295 (4.9)	6,065 (21.9)	1,820 (54.8)	225 (90.0)	< 0.001
Osteoporosis, n (%)	16,060 (14.7)	13,755 (15.8)	4,385 (15.8)	515 (15.5)	75 (30.0)	0.001
Osteoporosis with fracture, n (%)	985 (0.9)	955 (1.1)	340 (1.2)	50 (1.5)	5 (2.0)	0.078
Fluid or electrolyte disorder, n (%)	26,530 (24.2)	22,105 (25.4)	7,135 (25.7)	810 (24.4)	70 (28.0)	0.053
Connective tissue disease, n (%)	4,190 (3.8)	2,440 (2.8)	675 (2.4)	55 (1.7)	5 (2.0)	< 0.001
Hypothyroidism, n (%)	17,215 (15.7)	16,395 (18.8)	4,865 (17.5)	500 (15.1)	45 (18.0)	< 0.001
Local cancer, n (%)	0 (0.0)	2,390 (2.7)	1,350 (4.9)	315 (9.5)	40 (16.0)	< 0.001
Metastatic cancer, n (%)	0 (0.0)	1,595 (1.8)	650 (2.3)	200 (6.0)	20 (8.0)	< 0.001
Leukemia, n (%)	0 (0.0)	625 (0.7)	530 (1.9)	220 (6.6)	30 (12.0)	< 0.001
Lymphoma, n (%)	0 (0.0)	905 (1.0)	530 (1.9)	170 (5.1)	15 (6.0)	< 0.001

*OFS, Orthopedic Frailty Score*

The crude analyses indicated that a higher OFS was associated with a significantly increased risk of in-hospital mortality (OFS 0 vs. OFS 4: 0.5% vs 8.0%, p < 0.001), complications (OFS 0 vs. OFS 4: 26.1% vs 50.0%, p < 0.001), and FTR (OFS 0 vs. OFS 4: 0.4% vs 8.0%, p < 0.001). Patients with a high OFS also tended to require a longer hospital stay (OFS 0 vs. OFS 4: 4 days vs 6 days, p < 0.001) and incur higher costs for the hospital (OFS 0 vs. OFS 4: $15,804 vs $20,535, p < 0.001). The majority of complications became more prevalent as the OFS increased, except for cardiac tamponade, pulmonary embolism, wound infection, and implant infection (Table 3).

**Table 3:** Crude outcomes in hip fracture patients

	OFS 0 (N = 109,410)	OFS 1 (N = 87,115)	OFS 2 (N = 27,755)	OFS 3 (N = 3,320)	OFS 4 (N = 250)	P-value
In-hospital mortality, n (%)	550 (0.5)	1,350 (1.5)	875 (3.2)	115 (3.5)	20 (8.0)	< 0.001
Missing	35 (0.0)	20 (0.0)	0 (0.0)	0 (0.0)	0 (0.0)	
Complication, n (%)	28,590 (26.1)	33,745 (38.7)	13,860 (49.9)	1,810 (54.5)	125 (50.0)	< 0.001
Any cardiac complication, n (%)	1,025 (0.9)	1,645 (1.9)	870 (3.1)	115 (3.5)	5 (2.0)	< 0.001
Heart failure, n (%)	160 (0.1)	225 (0.3)	100 (0.4)	20 (0.6)	0 (0.0)	0.003
Cardiac arrest, n (%)	25 (0.0)	110 (0.1)	45 (0.2)	0 (0.0)	5 (2.0)	< 0.001
Cardiac tamponade, n (%)	0 (0.0)	15 (0.0)	5 (0.0)	0 (0.0)	0 (0.0)	0.414
Ventricular tachycardia, n (%)	680 (0.6)	985 (1.1)	540 (1.9)	80 (2.4)	0 (0.0)	< 0.001
Ventricular fibrillation, n (%)	30 (0.0)	110 (0.1)	45 (0.2)	5 (0.2)	0 (0.0)	0.002
Stroke, n (%)	825 (0.8)	940 (1.1)	360 (1.3)	35 (1.1)	0 (0.0)	0.001
Acute kidney injury, n (%)	11,510 (10.5)	15,055 (17.3)	6,820 (24.6)	900 (27.1)	70 (28.0)	< 0.001
Delirium, n (%)	2,565 (2.3)	4,360 (5.0)	1,785 (6.4)	225 (6.8)	35 (14.0)	< 0.001
Pulmonary embolism, n (%)	530 (0.5)	550 (0.6)	135 (0.5)	25 (0.8)	0 (0.0)	0.283
Deep vein thrombosis, n (%)	605 (0.6)	640 (0.7)	275 (1.0)	20 (0.6)	5 (2.0)	0.004
Embolism due to orthopedic device, n (%)	0 (0.0)	0 (0.0)	5 (0.0)	5 (0.2)	0 (0.0)	< 0.001
Sepsis, n (%)	1,555 (1.4)	1,675 (1.9)	670 (2.4)	85 (2.6)	10 (4.0)	< 0.001
Urinary tract infection, n (%)	10,220 (9.3)	11,495 (13.2)	4,280 (15.4)	665 (20.0)	30 (12.0)	< 0.001
Pneumonia, n (%)	6,135 (5.6)	7,085 (8.1)	3,625 (13.1)	465 (14.0)	55 (22.0)	< 0.001
Wound infection, n (%)	85 (0.1)	55 (0.1)	20 (0.1)	0 (0.0)	0 (0.0)	0.938
Implant infection, n (%)	10 (0.0)	20 (0.0)	0 (0.0)	0 (0.0)	0 (0.0)	0.669
Decubitus ulcer, n (%)	1,235 (1.1)	1,740 (2.0)	850 (3.1)	130 (3.9)	0 (0.0)	< 0.001
Failure-to-rescue, n (%)	490 (0.4)	1,195 (1.4)	765 (2.8)	115 (3.5)	20 (8.0)	< 0.001
Missing	10 (0.0)	0 (0.0)	0 (0.0)	0 (0.0)	0 (0.0)	
Length of stay, median [IQR]	4 [3.0–6.0]	5 [3.0–6.0]	5 [4.0–7.0]	5 [4.0–7.0]	6 [4.0–8.0]	< 0.001
Total cost of hospital stay ($), median [IQR]	15,804 [12,286–20,984]	16,451 [12,902–21,765]	17,526 [13,643–23,338]	18,034 [14,192–24,775]	20,535 [16,222–25,778]	< 0.001

*OFS, Orthopedic Frailty Score*

After adjusting for potential confounding, OFS 4 was associated with an almost ten-fold increase in the risk of in-hospital mortality [adjusted IRR (95% CI): 10.56 (4.02–27.7), p < 0.001], a 38% increased risk of complications [adjusted IRR (95% CI): 1.38 (1.03–1.85), p = 0.032], and an almost 11-fold increase in the risk of FTR [adjusted IRR (95% CI): 11.6 (4.36–30.9), p < 0.001], compared to OFS 0. Patients with OFS 4 also required a day and a half additional care [change in median length of stay (95% CI): 1.52 (0.97–2.08), p < 0.001] as well as cost approximately $5,200 more to manage [change in median cost of stay (95% CI): 5,166 (1,921–8,411), p = 0.002], compared to those with OFS 0. (Table 4).

**Table 4:** Adjusted outcomes in hip fracture patients

Outcome	OFS 0	OFS 1		OFS 2		OFS 3		OFS 4	
		IRR (95% CI)	P-value	IRR (95% CI)	P-value	IRR (95% CI)	P-value	IRR (95% CI)	P-value
In-hospital mortality	Reference	2.34 (1.80–3.04)	< 0.001	4.15 (3.08–5.59)	< 0.001	4.29 (2.64–6.97)	< 0.001	10.56 (4.02–27.7)	< 0.001
Complication	Reference	1.21 (1.17–1.25)	< 0.001	1.41 (1.36–1.47)	< 0.001	1.49 (1.37–1.61)	< 0.001	1.38 (1.03–1.85)	0.032
Failure-to-rescue	Reference	2.32 (1.75–3.08)	< 0.001	4.04 (2.93–5.56)	< 0.001	4.77 (2.90–7.85)	< 0.001	11.60 (4.36–30.9)	< 0.001

		Change in median (95% CI)	P-value	Change in median (95% CI)	P-value	Change in median (95% CI)	P-value	Change in median (95% CI)	P-value
Length of stay (days)	Reference	0.24 (0.19–0.29)	< 0.001	0.59 (0.50–0.68)	< 0.001	0.74 (0.47–1.01)	< 0.001	1.52 (0.97–2.08)	< 0.001
Total cost of hospital stay ($)	Reference	952 (781–1,124)	< 0.001	1,905 (1,640–2,171)	< 0.001	2,636 (2,078–3,194)	< 0.001	5,166 (1,921–8,411)	0.002

IRRs are calculated using Poisson regression models with robust standard errors. Change in median is calculated using quantile regression models. Missing values were managed using multiple imputation by chained equations. All analyses were adjusted for age, sex, race/ethnicity, income quartile, ischemic heart disease, cerebrovascular disease, peripheral vascular disease, uncomplicated diabetes mellitus, diabetes mellitus with chronic complications, end stage renal disease, dementia, connective tissue disease, hypothyroidism, liver disease, coagulopathy, obesity, fluid or electrolyte disorders, smoking status, chronic obstructive pulmonary disease, alcohol abuse, drug abuse, psychoses, depression, osteoporosis, osteoporosis with fracture, type of fracture, and type of surgery

*OFS, Orthopedic Frailty Score; IRR, incident rate ratio; CI, Confidence Interval*

## Discussion

This study, which makes use of the largest all-payer inpatient database in the United States, is the first that validates the results of previous national Swedish investigations into the association between the OFS and adverse outcomes. An elevated OFS was found to be associated with a significantly increased risk of complications, in-hospital mortality, as well as FTR. Patients with a high OFS also tended to require a longer hospital stay and were more costly to manage compared to their less frail counterparts. These relationships were present both before and after adjusting for confounding factors.

The results of the current investigation corroborate previous data that found an association between frailty, measured using the OFS, and all-cause mortality as well as all specific causes of mortality registered in the Swedish Cause of Death Register [5]. However, while the previous study focused on 30-day and 90-day mortality, the current analysis also found an association between the OFS and in-hospital mortality. This is also consistent with previous publications and systematic reviews, which found an association between frailty and an elevated risk of mortality [28–30], complications [28, 29], a longer hospital length of stay [1, 28, 30], and a higher cost of hospital stay in hip fracture patients [1, 30]. Of note, the OFS was able to detect these associations despite making use of a markedly simpler model compared to the previously listed studies [31–35].

It is well established that elderly individuals as well as those who have multiple medications, coexisting health conditions, and suffer from dementia, are highly susceptible to delirium in the postoperative period [36–39]. These vulnerabilities are common in patients with hip fractures [5–7, 40–46] particularly in those who are frail, making delirium a prevalent issue in orthopedic wards. Research has established a strong correlation between postoperative delirium and elevated mortality risk, especially in emergency settings [37]. To address this, several guidelines aimed at reducing the risk of delirium in geriatric patients have been proposed, which may warrant further evaluation and customization for frail patients [47–49]. Implementing such measures could potentially lower the risk of death following hip fracture surgery, especially in the most frail hip fracture patients.

As this and previous publications demonstrate, frail hip fracture patients are also associated with a substantial economic burden [1, 30], beyond even that of the non-frail hip fracture patient [3, 50, 51]. Concurrently, the rate of hip fractures in frail patients is rising due in large part to an increase in average life expectancy and cumulative comorbidities [9, 52–54]. Current predictions indicate that the annual number of hip fractures are expected to double by 2050 [13–15]. With between approximately 15% and 50% of hip fracture patients being classified as frail, this subgroup will take an increasingly more significant toll on both healthcare systems and the public that funds them as time progresses [4, 5, 21, 30, 55]. The utilization of risk stratification tools, including the OFS, affords healthcare providers the benefit of more efficiently allocating resources and expertise. This approach also enables the prompt identification of patients who exhibit an elevated risk of decline, thereby allowing for early intervention. Furthermore, orthogeriatric care has also been shown, through several systematic reviews, to be a cost-efficient method for decreasing length of stay, mortality, and even the risk of delirium among patients who have suffered a hip fracture [56–59]. Despite this, room for improvement remains. Presently, these models primarily consider chronological age when determining eligibility; however, it might be more appropriate to base it on the patients' degree of frailty instead [60–63].

This study draws upon the largest all-payer inpatient database in the United States, the NIS, which provides accurate national estimates for 97% of all inpatient hospitalizations, in order to analyze over 227,000 estimated hip fracture patients [20]. The OFS itself makes use of five dichotomous variables, all of which can easily be retrieved at the time of admission from the patient or the patient’s past medical records without additional blood tests or intraoperative data. The objective and easy-to-use nature of the OFS limits the risk of human error when used in clinical practice and reduces the effect of intercoder variability when used with ICD codes in retrospective datasets. However, it is important to note that the limitations of retrospective cohort studies apply, such as residual confounding and the risk of non-differential misclassification, particularly given the reliance on ICD-10 codes for many of the variables. The analyses were also restricted to the variables available in the NIS, which precluded analysis of long-term mortality, quality of life, and readmission, while it was not possible to adjust for potential perioperative and anesthesiologic confounders as well as early ambulation practices.

## Conclusion

Patients with an elevated Orthopedic Frailty Score display a substantially increased risk of mortality, complications, and failure-to-rescue. A high Orthopedic Frailty Score is also associated with a prolonged and more costly hospital stay. The Orthopedic Frailty Score, with its objective and readily obtainable variables, presents a desirable option for estimating frailty in hip fracture patients in the clinical setting. Future studies would benefit from investigating how the Orthopedic Frailty Score may be used to aid in patient management, such as preoperative optimization, surgical timing, as well as postoperative monitoring and rehabilitation.
